# Supplementary material for: Mechanisms governing the pioneering and redistribution capabilities of the non-classical pioneer PU.1
Source: Nat Commun. 2020 Jan 21;11:402. doi: 10.1038/s41467-019-13960-2 (PMC6972792; doi:10.1038/s41467-019-13960-2)
Supplement: Supplementary file 7 — Source data [file 41467_2019_13960_MOESM7_ESM.zip › Source_Data/FigureS2/FigureS2B_MotifScanOutput/KmeansCluster1/homerResults/motif46.similar.html]

motif46

## Information for motif46

A
C
G
T
A
G
T
C
G
T
C
A
A
G
C
T
C
T
A
G
C
G
T
A
A
C
T
G
G
T
C
A
C
T
G
A
A
G
T
C
  
Reverse Opposite:  

A
C
T
G
A
G
C
T
C
A
G
T
A
G
T
C
A
C
G
T
A
G
T
C
C
T
G
A
A
C
G
T
C
T
A
G
C
G
T
A
  

|  |  |
| --- | --- |
| p-value: | 1e-15 |
| log p-value: | -3.608e+01 |
| Information Content per bp: | 1.912 |
| Number of Target Sequences with motif | 80.0 |
| Percentage of Target Sequences with motif | 1.57% |
| Number of Background Sequences with motif | 188.2 |
| Percentage of Background Sequences with motif | 0.47% |
| Average Position of motif in Targets | 103.6 +/- 50.6bp |
| Average Position of motif in Background | 99.0 +/- 71.7bp |
| Strand Bias (log2 ratio + to - strand density) | 0.4 |
| Multiplicity (# of sites on avg that occur together) | 1.16 |
| Motif File: | file (matrix) reverse opposite |

### Similar de novo motifs found

|  |  |  |  |  |  |  |  |
| --- | --- | --- | --- | --- | --- | --- | --- |
| Rank | Match Score | Redundant Motif | P-value | log P-value | % of Targets | % of Background | Motif file |
| 1 | 0.856 | G C T A C T A G G A C T A G C T A G T C G C A T A G T C C T G A A G T C C A T G C T G A | 1e-15 | -34.950991 | 2.02% | 0.75% | motif file (matrix) |
